# Current Status of the Adoption, Utilization and Helpfulness of Health Information Systems in Korea

**DOI:** 10.3390/ijerph16122122

**Published:** 2019-06-15

**Authors:** Kyunghwa Seo, Han-Nah Kim, Hyeongsu Kim

**Affiliations:** 1 Research Institute for Healthcare Policy, Korean Medical Association, Seoul 04373, Korea; seokh@kma.org (K.S.); manna81@kma.org (H.-N.K.); 2 Department of Preventive Medicine, School of Medicine, Konkuk University, Seoul 05029, Korea

**Keywords:** health information system, Order Communication System, Electronic Medical Records, Picture Archiving and Communication System, utilization, helpfulness

## Abstract

As interest in medical informatization has been increasing steadily worldwide, the adoption of health information systems (HISs) in medical institutions is essential. In this study, we intended to identify users’ adoption, utilization and helpfulness derived from HISs to determine the status of medical informatization by using 2016 Korean Physician Survey data. A total of 8564 members responded to the 2016 survey, we extracted the data of 6949 HISs related part for this study. As a result, about 68% of the self-employed physicians adopted the Order Communication System and Electronic Medical Records, while only one-third adopted the Picture Archiving and Communication System. Overall, the proportions of utilization and helpfulness of HISs were higher for females; for employed physicians or professors; for physicians working in hospitals; and for those specializing in surgical medicine. As applying information technology to the medical environment becomes more important, HIS will become a necessary requirement and the demand for information systems considering user and patient oriented information environments will be increasing. Therefore, it is necessary to discuss the HISs use environment considering not only technical aspects but also users’ or patients’ viewpoints. In that respect, this study will give a meaningful evidence of HIS related policies.

## 1. Introduction

Health information systems (HISs) are widely referred to as the mutual interaction of people, processes, and technologies to provide information necessary for the improvement of healthcare service quality [1,2]. More specifically, HISs are a tool for collecting, storing, analyzing, and utilizing various data, including patient medical information, generated in medical institutions [3,4]. Thus the HISs are one of the elements of the healthcare system [2]. 

HISs are becoming increasingly important in light of the rapid generation and accumulation of medical information and the increasing demand for healthcare. Indeed, HIS data are now being processed as big data [5]. The interest in a healthcare industry based on information and communications technology (ICT) is also increasing [6] since the arrival of the Fourth Industrial Revolution. In addition, the Korean government raised the budget for medical informatization projects (healthcare big data and the utilization of HISs in precision medicine) in 2019 [7]. With the rapid development of Korean ICT, adoption or use of information systems in the healthcare sector has become more common than ever. As the excellence of Korean health information system or related technology is acknowledged abroad, other countries are interested in adopting Korean HISs. In fact, government affiliated hospitals or organizations have been signed an MOU (Memorandum of Understanding) to export HISs abroad [8]. Considering the meaning of informatization refers to ‘use of information technology resources in organizations’ [9], changes in perceptions and paradigms of medical informatization are taking place across the world. For example, in the United States, the government actively encourages the introduction of the health information technology (HIT) [10] and expert groups including the American Medical Association are also promoting expansion of HISs to improve treatment and patient safety in their clinical settings [11]. Now, the perception that it is not possible to run the complex healthcare business without using HISs is widespread [11].

This trend towards medical informatization is believed to have had a positive impact on organizational operation: for example, HIS adoption and use has led to an increase in task efficiency [12]. In the past, HISs were just adopted without any recognition of impact on patient diagnosis and hospital tasks [13]. Moreover, HISs were not utilized, or used very little, despite widespread recognition of the importance of medical informatization at that time [14]. However, recent empirical evidence has confirmed that medical informatization due to HIS adoption and utilization has had a positive effect on the care delivery process and medical service outcomes, as well as overall hospital management performance [15,16,17,18]. Unlike the above-mentioned, some studies have identified the negative impact of the HISs adoption. The burden of cost, disruption of work flow, decrease in revenues, and risk of patient privacy violations are the potential disadvantages of HISs adoption or use [19]. Similarly, additional costs including manpower may be needed for HIS maintenance and management, and patient privacy issues may be time-consuming to address as well [20]. Despite of these negative aspects, considering the overall benefits of HIS, medical informatization has become a trend, and HIS adoption has become a requirement rather than a choice for many medical institutions in Korea.

Besides that, there are many studies related to the HISs as medical informatization spreads rapidly. Particularly, research in Korea has tended to focus on the technological aspects of HIS design and realization [21,22,23,24,25,26,27], privacy protection and system security [26,28,29,30,31], system standardization [32,33,34] and, more recently, the integration of HISs [22,35,36,37,38,39]. Though there are many forms of relevant research, few studies related to the users’ attitudes towards or perceptions of the HISs have been conducted. Therefore, we sought to determine the utilization of HISs among practitioners in clinical sites as well as the extent to how much the HISs help to support practitioners. More specifically, we intended to examine the status of HIS adoption within medical institutions as well as the utilization and helpfulness of HISs according to various respondent parameters.

## 2. Materials and Methods

### 2.1. Data and Study Population

This study used a part of the 2016 Korean Physician Survey (KPS) data. The 2016 KPS was a web-based cross-sectional survey conducted by the Research Institute for Healthcare Policy (RIHP) of the Korean Medical Association (KMA). Of 108,870 physicians who registered their basic information in database in KMA, through the stratified quota sampling 61,983 ones were sent a questionnaire and a total of 8564 members participated in 2016 KPS. They were asked to evaluate the work environment and status, healthcare system/policy awareness and assessment, adoption status and users’ attitudes on HISs, occupational satisfaction, lifestyle habits/health status and future plan through a structured questionnaire. Of a total 8564 members responding to the 2016 survey, we extracted the HISs related part of the 2016 KPS data for this study.

In this study, we included practicing physicians working in medical institutions in Korea. However, physicians working in dental clinics/hospitals, oriental medicine clinics/hospitals, and public health institutions (health center, health subcenter, national health center and county hospital), military hospitals, correctional institutions, long-term hospitals, and other medical-service-related institutions were excluded because they did not represent the characteristics of general medical institutions. Also, we excluded medical residents who were still undergoing training or were in military service. Thus, the data of 6949 self-employed physicians, employed physicians, and medical school faculty were analyzed.

### 2.2. Definition of HIS

HISs are typically classified into three categories according to their primary tasks in the hospital: administration, diagnosis and diagnostic support, and management [14]. In this study, we focused on the HISs related to the work of medical care and medical support. The three types of HISs are defined as follows.

- Order Communication System (OCS): This system allows various lab and imaging tests or treatment services to be ordered instantly through networks.
- Electronic Medical Record (EMR): This system is a database containing computerized forms of patients’ paper-based medical charts; all information on these charts is entered, managed, and stored to enable physicians to access these records without restrictions of time or space.
- Picture Archiving and Communication System (PACS): This system concerns the acquisition, storage, transmission, and display of medical images digitally from various examination devices such as X-rays, computed tomography (CT), and magnetic resonance imaging (MRI).

### 2.3. Variables and Analysis

The key variables were gender; age; employment status; specialty; type of institution; practice location; and the status of adoption, utilization and helpfulness of HISs (OCS, EMR, and PACS). Specialty was categorized as internal medicine, surgical medicine, and medical assistance specialty. The internal medicine category included internal medicine, neurology, psychiatry, pediatrics, dermatology, phthisiology, rehabilitation, and family medicine; surgical medicine included general surgery, orthopedics, neurosurgery, plastic surgery, obstetrics and gynecology, ophthalmology, otolaryngology, urology, emergency medicine; and the medical assistance specialty included anesthesia, radiology, radiation oncology, pathology, laboratory medicine, preventive medicine, nuclear medicine, and occupational and environmental medicine. Medical institutions with less than 30 beds and more than 30 beds were classified as clinics and hospitals, respectively. Capital area includes Seoul, Gyeonggi, and Incheon; metropolitan cities included Busan, Daegu, Gwangju, Daejeon, and Ulsan; and all other areas were classified as regional areas. In the analysis of factors related to the utilization and helpfulness, ‘*n*’ refers to the number of people who responded with ‘use sometimes’ or ‘use always’ for utilization, and ‘helpful’ or ‘very helpful’ for helpfulness on the five-point Likert scale.

We analyzed adoption rate of HIS, utilization and helpfulness of HISs to determine status of adoption and physicians’ overall attitudes towards HIS in Korea according to various respondent parameters. In terms of the adoption status on HISs, it should be determined at the level of the medical institution. Given that the target subjects of 2016 KPS were physicians, we analyzed the status of HISs adoption only for self-employed physicians. This was because self-employed physicians are considered to represent their institutions, so their individual-level answers were deemed appropriate. In aspects of utilization and helpfulness of HISs, which were individual-level attitudes, we performed the analysis only on individuals who responded that they had adopted an HIS in their clinical setting. The chi-square test was performed to verify the differences in adoption, utilization and helpfulness of the HISs by respondent characteristics. SAS 9.4 (SAS Institute, Cary, NC, USA) was used for the statistical analysis.

### 2.4. Ethics Statement

This study has been exempted from review (Task No. 7001355-201804-E-070) by the Institutional Review Board of Konkuk University. Only a part of the processed secondary data from 2016 KPS data has been used, excluding personal identification and sensitive information.

## 3. Results

### 3.1. Characteristic of the Study Population

Of a total study population of 6849 individuals, the majority were men (83.7%) and the most common age group was 40–59 years (70.0%) (Table 1). The largest proportion of employment status was self-employed (48.7%), followed by employed (34.1%). As for specialty, there was an order of internal medicine (50.7%), surgical medicine (39.6%) and medical assistant specialty (9.6%). 

### 3.2. Current Status on Adoption of HIS

The findings for the status of HIS adoption are summarized in Table 2. About 68% of the self-employed physicians had adopted the OCS and EMR within their medical institutions, whereas only 32.9% had adopted a PACS. Overall, physicians in the lower age groups exhibited higher proportions of HIS adoption—in fact, over 80% of self-employed physicians below the age of 50 had adopted the OCS and EMR, while only 50% of respondents over the age of 60 had done so. Physicians working at hospital-level medical institutions had significantly higher adoption rates for OCS (85.1%), EMR (75.0%), and PACS (63.3%) than did physicians working at clinic-level medical institutions. The OCS adoption rate did not significantly differ by specialty and practice location. However, the adoption rate of EMR was higher among self-employed physicians specializing in internal medicine (70.0%) and that of PACS was higher among those with a medical assistant specialty (47.9%). These two systems both had relatively higher adoption rates in areas outside of capital areas. 

### 3.3. Utilization and Helpfulness of HISs

#### 3.3.1. OCS

Over 80% of the total 5631 respondents working in environments with an OCS were evaluated positively concerning the utilization and helpfulness of OCS (Table 3). There were significant differences for all variables in utilization, and the same was true for all variables with exception of specialty in helpfulness. 

For the utilization of OCS, females (87.6%) had a significantly higher utilization proportion (“use sometimes” or “use always”) than did males (83.2%). Also, the utilization proportion was higher among those in the lower age groups: individuals aged under 40 (94.7%) were using OCS more compared to those aged over 60 (69.1%). The utilization proportions of OCS among medical school faculty and employed physicians were 94.7 and 91.7 percent, respectively, which were about 20 percent higher than for the self-employed physicians. More than 80 percent of the respondents among those specializing in internal medicine or surgical medicine answered that they were using the OCS in their clinical settings. Meanwhile, physicians working in hospital-level medical institution (94.1%) and in the capital area (86.0%) exhibited higher utilization proportions for OCS compared to other groups. 

The results for helpfulness of OCS were mostly similar to that of utilization except that the proportions (“helpful” or “very helpful”) were about 2–3 percentage points lower than were those for utilization. 

#### 3.3.2. EMR

Among the 5463 respondents who reported adopting EMR, 87.8 percent had used EMR “sometimes” or “always” and 81.1% thought EMR was “helpful” or “very helpful” (Table 4). 

Regarding the results for EMR utilization, females (92.5%) had a higher proportion than males (86.9%). Furthermore, 95.9% of respondents below age 40 were using EMR, while only 76.2% of individuals aged over 60 were. Among employed physicians and medical school faculty, more than 90 percent were using EMR, while the utilization proportion among self-employed physicians was below 80 percent. Also, the percent of respondents whose specialty was surgical medicine was nearly 90%, which were higher than those specializing in internal medicine (86.9%) or a medical assistant specialty (85.8%). Over 96 percent of respondents working in hospital-level medical institutions were using EMR, which was significantly more than those in clinic-level medical institutions. As for practice location, the *p*-values were higher than significance level (0.05), but larger regions tended to have higher utilization proportions of EMR (in the order of capital area, 89.3%; metropolitan cities, 88.0%; and regional areas, 86.8%). 

As for the analysis of EMR helpfulness, females (86.5%) responded more positively than males (80.0%). Moreover, 93.1% of respondents under the age of 40 perceived that usage of EMR was helpful in clinical settings. Employed physicians (88.7%) and medical school faculty (87.9%) also thought EMR was more useful than self-employed physicians (70.9%). Additionally, helpfulness proportions of physicians working in hospital-level medical institutions (89.4%) or capital area (82.9%) were higher than the other groups. 

#### 3.3.3. PACS

A total of 4308 physicians reported adopting PACS in their clinical settings, of which 93.9% indicated using it and 93.8% reported having it helpful for medical care activities (Table 5). Most of the results showed significant differences in their utilization and helpfulness proportions by respondents’ characteristics. More specifically, female (95.6%) physicians were using PACS a little more than males (93.6%). Over 90% of respondents under the age of 50 years and employed physicians/medical school faculty were using PACS. The utilization proportion of all specialties was at least 92 percent, with the surgical specialty having the highest proportion (95.1%). There was a 10 percentage point difference in positive response proportion between physicians who working in clinic-level medical institutions (87.9%) and those who working in hospital-level medical institutions (96.5%). However, as for region, there was no significant difference within groups. 

As for helpfulness, the majority of the respondents perceived that usage of PACS was more helpful to practice. The pattern of responses did not differ much from that for utilization by variables except practice location. The respondents working in the capital area (95.1%) had statistically significant higher proportion of PACS utilization compared to other areas. Overall, physicians aged 60, self-employed physicians, and physicians working in clinic-level medical institutions had lower ratios for utilization of PACS and perception of it being helpful to practice, although the ratios were still above 80%. 

## 4. Discussion

Changes in perceptions and paradigms of medical informatization are taking place across the world and medical informatization is also a more common issue in Korea. Note that the meaning of informatization refers to ‘use of information technology resources in organizations’ [9] in this study. This study was intended to provide the current status of Korea’s medical informatization by determining adoption status on HISs in medical institutions, overall users’ attitudes toward HISs and its related factors. We analyzed the adoption status, utilization, and helpfulness of HISs for their medical care activities among physicians according to their characteristics. We focused on the OCS, EMR, and PACS as representative of HISs. 

The analysis of HIS adoption status, which was conducted among the 3337 self-employed physicians (of the 6849 total respondents), revealed that about 68% of respondents had adopted OCS or EMR, whereas only 33% had adopted PACS. This was rather different from the findings of a survey on hospital informatization conducted by the Korea Health Industry Development Institute in 2015. That survey was conducted one year prior to the KPS, and while the adoption rates in the clinic-level medical institutions (OCS, 69.1%; EMR, 61.4%; PACS, 29.3%) were similar to those found in our study, the adoption rates for hospital-level medical institutions were higher than in our study by about ten percentage points (OCS, 91.9%; EMR, 83.9%; PACS, 85.4%) [40].

According to the results of utilization and helpfulness of HISs, over 80% were using the OCS and EMR and perceived them as helpful, although with lower rates of perceived helpfulness for OCS and EMR, when compared to utilization. For the PACS, on the other hand, ratios for utilization and helpfulness were 93.9% and 93.8%, respectively, suggesting that this system was highly useful and considered helpful in clinical setting. When looking at the analysis results by respondent characteristics, the utilization and helpfulness of all three types of HISs exhibited consistent trends according to gender, age, status of employment, type of institution, specialty, and practice location. More specifically, rates of utilization and helpfulness of HISs were higher for females than for males, and for employed physicians or professors than for self-employed physicians; for physicians working in hospital-level medical institutions than for those working in clinic-level medical institutions; and for those specializing in surgical medicine than for those in other specialties. Regarding practice location, while we found that utilization and helpfulness were higher in the capital area for all three HISs, the difference was not significant for utilization of the PACS. The result that younger individuals are more likely to use HISs is supported by the findings of O’Donnell et al [41], which systemically reviewed international literature on the attitudes of primary care physicians (PCP) towards EMR. They reported that younger, computer-literate physicians, based in large/multi-group practices, were more likely to be positively inclined to EMR use than older, less-skilled physicians based in solo practices. In addition, adequate training, policies, procedures and financial factors from start-up costs to the resources required by ongoing use favorably impacted on PCPs’ views on EMR adoption and implementation.

Furthermore, we identified that the employment status of respondents is also a crucial factor related to utilization and helpfulness of HISs. This is supported by the findings of Kokkonen et al. [42], in which the HIS utilization rate of employed physicians was higher than that of self-employed. The HIS adoption proportion for hospital-level medical institutions was higher than was that for clinic-level medical institutions in this study. Similar results were obtained in an NCHS Data Brief [16] and an ONC Data Brief [43] of the US, wherein the HIS adoption rate varied with the number of physicians in a medical institution; these findings indicate that there is a difference in HIS adoption rate according to the scale of the medical institution. Meanwhile, our result of a higher proportion for utilization and helpfulness of HISs among surgeons was supported by Frazee et al. [18]. They also found positive recognition of electronic health records (a type of HIS) usage among surgeons, although their study did not involve group comparison. We suggest that these findings result from the fact that primary care physicians typically perform medical care activities based in offices, whereas surgeons perform their activities in various areas of the medical institution, including their offices, operating rooms, intensive care units, and emergency rooms, all of which indirectly supports the results of this study. However, it did not align with the findings of Kokkonen et al. [42], who found that the utilization and helpfulness of EMR among surgical medicine physicians were not higher compared to among other specialties. 

This study has several limitations. First, our data lacked comprehensive coverage of physicians’ attitude and awareness towards HISs and the overall status of medical informatization in Korea. HISs were not the main focus of the 2016 KPS, and few significant conclusions can be drawn from an analysis of only a few questions. Secondly, we surveyed at the individual level of healthcare service providers to obtain data on HISs use status. While we assessed HIS adoption among all the respondents, we cannot precisely determine adoption status at the hospital-level of the medical institution using their data. To correct for this issue, we analyzed the adoption status only among self-employed physicians. Finally, we did not account for possible differences in the level of knowledge of HISs among respondents, which could have led to differences in actual adoption status. Therefore, these limitations require consideration when generalizing these results of our study and comparing them to the results of other surveys. 

## 5. Conclusions

Throughout Korea, the majority of medical institutions have implemented HISs, over 90% of hospital-level medical institutions and 75% of clinic-level medical institutions have adopted OCS and EMR as of 2017. As applying information technology to the medical environment becomes more important, HISs will become a necessary requirement rather than a choice and the demand for information systems considering user and patient oriented information environments will be increasing. Moreover, HISs will serve as critical tools contributing to enhance working conditions as well as quality of medical services through medical informatization led by users and patients, and to establish a patient-oriented healthcare environment. However, most HISs of Korean medical institutions are designed to focus on technical aspects of raising work efficiency and aiding decision making in clinical settings rather than users’ or patients’ perceptions. Considering secure storage of patients’ information or user-friendly interfaces, the more sensitive factors including security and standardization must be considered in adopting and using the HISs. Therefore, it is necessary to discuss the HISs use environment considering not only technical aspects but also users’ or patients’ viewpoints as well as sensitive factors. Despite of some limitations, it is expected that this study will give meaningful evidence based on the users’ viewpoints to inform HIS related policies. In the future, more detailed research should be conducted with well-structured questionnaires to overcome the limitations of this study.

## Figures and Tables

**Table 1:** Characteristics of respondents.

		N	%
Gender	Male	5731	83.7
	Female	1118	16.3
Age (yrs, 47.55 ± 9.75)	Under 40	912	13.3
	40–49	2557	37.3
	50–59	2240	32.7
	Over 60	1140	16.6
Employment status	Self-employed	3337	48.7
	Employed	2338	34.1
	Medical school faculty	1174	17.1
Specialty	Internal medicine	3474	50.7
	Surgical medicine	2715	39.6
	Medical assistant specialty	660	9.6
Type of institution	Clinic-level	3635	53.1
	Hospital-level	3214	46.9
Ownership of institution	Public	586	8.6
	Private	6263	91.4
Practice location	Capital area	3369	49.2
	Metropolitan cities	1681	24.5
	Regional areas	1799	26.3
**Total**		6849	100.0

**Table 2:** Current status of health information system (HIS) adoption among self-employed physicians.

		Total	OCS		EMR		PACS	
			*n* (%)	*p*-Value	*n* (%)	*p*-Value	*n* (%)	*p*-Value
Gender	Male	2297	2044 (68.2)	0.385	2049 (68.4)	0.547	1032 (34.4)	<0.0001
	Female	340	224 (65.9)		227 (66.8)		65 (19.1)	
Age	Under 40	65	57 (87.7)	<0.0001	55 (84.6)	<0.0001	26 (40.0)	<0.0001
	40–49	46	814 (86.1)		723 (76.4)		458 (48.4)	
	50–59	1530	993 (64.9)		1035 (67.7)		460 (30.1)	
	Over 60	796	404 (50.8)		463 (58.2)		153 (19.2)	
Specialty	Internal medicine	1849	1263 (68.3)	0.767	1294 (70.0)	0.047	538 (29.1)	<0.0001
	Surgical medicine	1298	880 (67.8)		855 (65.9)		468 (36.1)	
	Medical assistant specialty	190	125 (65.8)		127 (66.8)		91 (47.9)	
Type of Institution	Clinic-level	3149	2.108 (66.9)	<0.0001	2135 (67.8)	0.039	978 (31.1)	<0.0001
	Hospital-level	188	160 (85.1)		141 (75.0)		119 (63.3)	
Practice Location	Capital area	914	596 (65.2)	0.109	594 (65.0)	0.043	259 (28.3)	0.001
	Metropolitan cities	807	555 (68.8)		566 (70.1)		266 (33.0)	
	Regional areas	1616	1117 (69.1)		1116 (69.1)		572 (35.4)	
**Total**		3337	2268 (68.0)		2276 (68.2)		1097 (32.9)	

Unit: person (%). HIS = Health Information System, OCS = Order Communication System, EMR = Electronic Medical Record, PACS = Picture Archiving and Communication System.

**Table 3:** Factors related with the utilization and helpfulness of OCS.

		Respondents Who Have Adopted OCS	Utilization		Helpfulness	
			*n* (%)	*p*-Value	*n* (%)	*p*-Value
Gender	Male	4661	3879 (83.2)	0.001	3766 (80.8)	<0.0001
	Female	970	850 (87.6)		842 (86.8)	
Age	Under 40	875	829 (94.7)	<0.0001	815 (93.1)	<0.0001
	40–49	2365	2153 (91.0)		2072 (87.6)	
	50–59	1666	1246 (74.8)		1233 (74.0)	
	Over 60	725	501 (69.1)		488 (67.3)	
Employment status	Self-employed	2268	1611 (71.0)	<0.0001	1571 (69.3)	<0.0001
	Employed	2213	2029 (91.7)		1977 (89.3)	
	Medical school faculty	1150	1089 (94.7)		1060 (92.2)	
Specialty	Internal medicine	2803	2363 (84.3)	0.003	2271 (81.0)	0.229
	Surgical medicine	2248	1907 (84.8)		1852 (82.4)	
	Medical assistant specialty	580	459 (79.1)		485 (83.6)	
Type of Institution	Clinic-level	2529	1810 (71.6)	<0.0001	1775 (70.2)	<0.0001
	Hospital-level	3102	2919 (94.1)		2833 (91.3)	
Practice Location	Capital area	1685	1449 (86.0)	0.001	1416 (84.0)	0.011
	Metropolitan cities	1404	1195 (85.1)		1148 (81.8)	
	Regional areas	2542	2085 (82.0)		2044 (80.4)	
**Total**		5631	4729 (84.0)		4608 (81.8)	

Unit: person (%). OCS = Order Communication System.

**Table 4:** Factors related with the utilization and helpfulness of EMR.

		Respondents Who Have Adopted EMR	Utilization		Helpfulness	
			*n* (%)	*p*-Value	*n* (%)	*p*-Value
Gender	Male	4549	3954 (86.9)	<0.0001	3640 (80.0)	<0.0001
	Female	914	845 (92.5)		791 (86.5)	
Age	Under 40	825	791 (95.9)	<0.0001	768 (93.1)	<0.0001
	40–49	2193	2039 (93.0)		1861 (84.9)	
	50–59	1667	1384 (82.5)		1247 (74.4)	
	Over 60	768	585 (76.2)		555 (72.3)	
Employment status	Self-employed	2276	1776 (78.0)	<0.0001	1613 (70.9)	<0.0001
	Employed	2068	1938 (93.7)		1835 (88.7)	
	Medical school faculty	111	1085 (97.0)		983 (87.9)	
Specialty	Internal medicine	2739	2381 (86.9)	0.006	2188 (79.9)	0.057
	Surgical medicine	2162	1936 (89.6)		1785 (82.6)	
	Medical assistant specialty	562	482 (85.8)		458 (81.5)	
Type of Institution	Clinic-level	2511	1962 (78.1)	<0.0001	1793 (71.4)	<0.0001
	Hospital-level	2952	2837 (96.1)		2639 (89.4)	
Practice Location	Capital area	1616	1443 (89.3)	0.062	1339 (82.9)	0.090
	Metropolitan cities	1379	1213 (88.0)		1114 (80.8)	
	Regional areas	2468	2143 (86.8)		1978 (80.2)	
**Total**		5463	4799 (87.8)		4431 (81.1)	

Unit: person (%). EMR = Electronic Medical Records.

**Table 5:** Factors related with the utilization and helpfulness of PACS.

		Respondents Who Have Adopted EMR	Utilization		Helpfulness	
			*n* (%)	*p*-Value	*n* (%)	*p*-Value
Gender	Male	3556	3327 (93.6)	0.033	3319 (94.3)	0.013
	Female	752	719 (95.6)		720 (95.7)	
Age	Under 40	783	748 (95.5)	<0.0001	752 (96.0)	<0.0001
	40–49	1952	1863 (95.4)		1857 (95.1)	
	50–59	1112	1028 (92.5)		1016 (91.4)	
	Over 60	461	407 (88.3)		414 (89.8)	
Employment status	Self-employed	1097	978 (89.2)	<0.0001	979 (89.2)	<0.0001
	Employed	2039	1928 (94.6)		1931 (94.7)	
	Medical school faculty	1172	1140 (92.3)		1129 (96.3)	
Specialty	Internal medicine	1955	1820 (93.1)	0.018	1813 (92.7)	0.040
	Surgical medicine	1807	1719 (95.1)		1711 (94.7)	
	Medical assistant specialty	546	507 (92.9)		515 (94.3)	
Type of Institution	Clinic-level	1291	1135 (87.9)	<0.0001	1146 (88.8)	<0.0001
	Hospital-level	3017	2911 (96.5)		2893 (95.9)	
Practice Location	Capital area	1309	1233 (94.2)	0.331	1245 (95.1)	0.003
	Metropolitan cities	1077	1019 (94.6)		1018 (94.5)	
	Regional areas	1922	1794 (93.3)		1776 (92.4)	
**Total**		4308	4046 (93.9)		4039 (93.8)	

Unit: person (%). PACS = Picture Archiving and Communication System.
